# Safe femoral condyle range for the reverse Rigidfix femoral fixation device in anterior cruciate ligament reconstruction

**DOI:** 10.1186/s12891-022-05250-8

**Published:** 2022-03-25

**Authors:** Zhongliu Luo, Yong Hu, Qingmin Han, Zhi Gao, Songmiao Cheng

**Affiliations:** 1 The Third Clinical Medical School, Guangzhou University of Chinese Medicine, No. 232, Outer Ring East Road, Guangzhou University City, Panyu District, Guangzhou City, Guangdong Province China; 2 Department of Knee Joint Sports Injury, Sichuan Provincial Orthopedic Hospital, No. 132, West Section, First Ring Road, Sichuan Province Chengdu, China

**Keywords:** Anterior cruciate ligament reconstruction, Rigidfix, Arthroscopy, Cross-pin fixation, Anteromedial portal

## Abstract

**Background:**

To determine the characteristics of cross-pin protrusion in patients treated with the reverse Rigidfix femoral fixation device for femoral tunnel preparation through the anteromedial portal in Arthroscopic anterior cruciate ligament reconstruction (ACLR), analyse the reasons for this outcome, and identify safety hazards of this surgical technique for improvement.

**Methods:**

A retrospective analysis of patients who underwent ACLR using this technology at our hospital in 2018 was conducted. Patients with and without cross-pin protrusion were included in the protrusion positive and negative groups, respectively. The sex, age and imaging characteristics of the patients with cross-pin protrusion were identified, and the reasons for cross-pin protrusion were analysed.

**Results:**

There were 64 and 212 patients in the protrusion positive and negative groups, respectively. The proportion of cross-pin protrusion cases was 23.19% (64/276). There was a significant difference in the ratio of males to females (*P* < 0.001, *χ2* = 185.184), the mediolateral femoral condyle diameter (protrusion positive group, 70.59 ± 2.51 mm; protrusion negative group, 82.65 ± 4.16 mm; *P* < 0.001, *t* = 28.424), and the anteroposterior diameter of the lateral femoral condyle (protrusion positive group, 58.34 ± 2.89 mm; protrusion negative group, 66.38 ± 3.53 mm; *P* < 0.001, *t* = 16.615). The cross-pins did not penetrate the lateral femoral condyle cortex in patients with a mediolateral femoral condyle diameter ≥ 76 mm, but the cross-pins definitely penetrated the cortex when the diameter was ≤ 70 mm. The cross-pins did not penetrate when the anteroposterior lateral femoral condyle diameter was ≥ 66 mm, but the cross-pins definitely penetrated it when the diameter was ≤ 59 mm.

**Conclusion:**

The patients with cross-pin protrusion after reverse Rigidfix femoral fixation treatment to prepare the femoral tunnel through the anteromedial portal in ACLR were mainly females with small femoral condyles. For patients with a mediolateral femoral condyle diameter ≥ 76 mm and an anteroposterior lateral femoral condyle diameter ≥ 66 mm, there is no risk of cross-pin protrusion, so this technique can be used with confidence.

**Levels of evidence:**

III.

**Supplementary Information:**

The online version contains supplementary material available at 10.1186/s12891-022-05250-8.

## Introduction

**Fig. 1:**
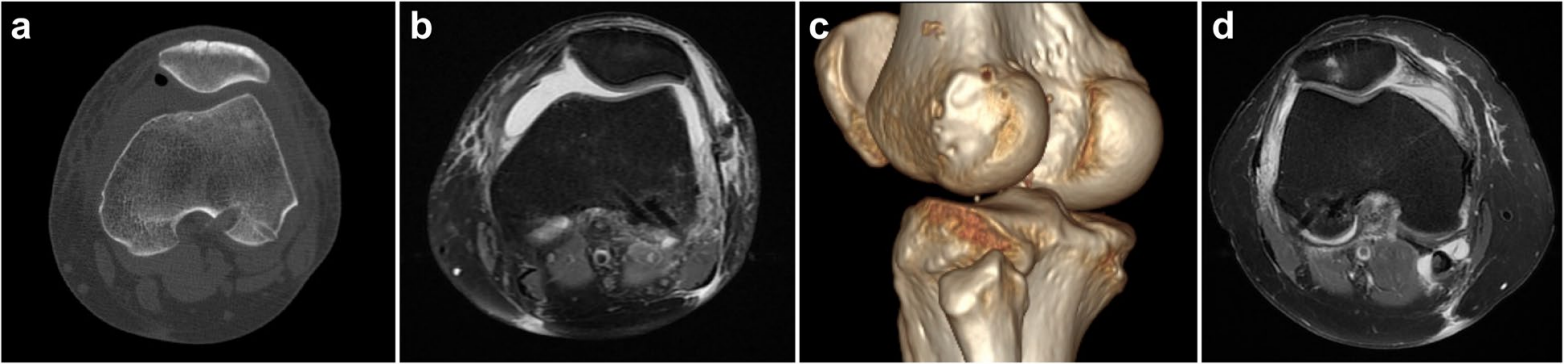
**a** Cross-pin penetration of the femoral condyle cortex on postoperative CT. **b** Cross-pin penetration of the femoral condyle cortex on postoperative MRI. **c** Cross-pin penetration of the femoral condyle cortex on postoperative three-dimensional CT. **d** Cross-pin penetration of the femoral condyle cortex and injury of the posterolateral joint capsule

Arthroscopic anterior cruciate ligament reconstruction (ACLR) is very important for anterior cruciate ligament (ACL) rupture; it can effectively restore the stability of the knee joint and reduce secondary injury of the meniscus and cartilage caused by ACL rupture [1]. Intraoperative treatment of the femur can be challenging and critical to proper ACLR. The focus of the intraoperative treatment of the femur is femoral tunnel positioning and the selected method of graft fixation. Pearle et al. [2] reported that the IDEAL point is the best femoral tunnel positioning point in single-bundle ACLR; this point is located in the anterior (high) and proximal (deep) region of the footprint, is anatomical (within the femoral footprint), replicates the low tension-flexion pattern of the native ACL throughout the range of flexion and extension, and is closer to the position of the isometric point. Among cross-pin fixation devices, Rigidfix (Depuy Mitek, Norwood, MA) is ​​the most widely used. The Rigidfix femoral fixation device requires femoral tunnel positioning using the transtibial (TT) technique, and the cross-pins are inserted laterally. However, with this technique, the choice of the femoral tunnel position is relatively limited, and damage to the lateral knee muscles can easily occur, leading to dysfunction of the knee extensor muscles and reducing the range of motion of the knee joint in the early postoperative period [5]. If the needle insertion angle is not properly selected when making cross-pin tunnels, iatrogenic neurovascular injury may even occur [6, 7]. In a study by Hu et al. [8], the use of the reverse Rigidfix femoral fixation device to prepare the femoral tunnel through the anteromedial (AM) portal reduced iatrogenic injury, demonstrating the advantage of using the AM portal technique for femoral tunnel positioning. This method is clinically reliable and effective. However, during the 3-month follow-up process, a small number of patients had discomfort near the popliteal tendon femoral insertion point during joint movement, and the range of movement was reduced in the early postoperative period. After postoperative MRI and CT examinations, the cross-pins had penetrated the lateral femoral condylar cortex (Fig. 1) due to irritation of the popliteal tendon and lateral joint capsule (Fig. 1d).

The reason for cross-pin protrusion in the use of this technique has not been previously investigated. The aim of this study was to determine the characteristics of patients with cross-pin protrusion after ACLR using this technique, to analyse the reasons for this outcome, and to determine the potential safety hazards of this surgical technique in order to enable further improvements.

## Methods

In this retrospective study, written informed consent was obtained from all patients included, and all patients provided valid consent to participate. All methods of this study were carried out in accordance with relevant guidelines and regulations. This study design was reviewed and approved by Sichuan Provincial Orthopedic Hospital Ethics Committee (approval No. KKY − 2018–03-01). A retrospective analysis of collected data from our hospital database was conducted. All patients who underwent ACLR with reverse Rigidfix femoral fixation to prepare the femoral tunnel through the AM portal and fix the femur with cross-pins (Rigidfix) between January 1, 2018, and December 31, 2018, were included in the study. In all patients, hamstring tendons were used as grafts for single-bundle ACLR, and review MRI and CT examinations were completed within 1 week after surgery. Patients were limited to patients with closed epiphyseal lines. To minimize the influence of abnormal femoral condyle morphology on the study, patients with severe arthritis of the femoral condyle combined with bone hyperplasia or deformation (Kellegren Lawrence grade II and above) and patients with femoral condyle fractures were excluded.

Preoperatively, all patients had sustained a knee injury resulting in ACL rupture diagnosed with clinical examination and MRI. All procedures were performed by 1 of 3 experienced surgeons (Y.H., Z.G., S.C.). All patients underwent the same postoperative rehabilitation protocol. Clinical evaluation of the outcome was conducted using the Lysholm score, the Tegner score and an objective side-to-side measurement of knee laxity using a Rolimeter (Aircast, Europa) at the 12-month follow-up.

According to whether cross-pin protrusion was observed with MRI or CT, patients with and without cross-pin protrusion were included in the protrusion positive and negative groups, respectively.

**Fig. 2:**
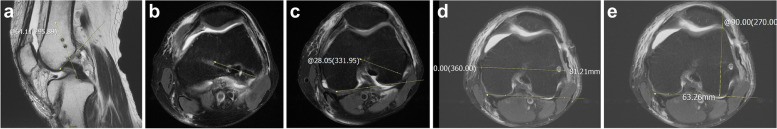
**a** Measure the angle between the line of the midpoints of the two cross-pins and Blumensaat's line on the postoperative image. **b** Mark the direction of the cross-pin on the postoperative image. **c** Measure the angle between the direction of the cross-pin and the line along the lowest point of the posterior side of the medial and lateral femoral condyles on the postoperative image. **d** Measure the mediolateral diameter of the femoral condyle on the image. **e** Measure the anteroposterior diameter of the lateral femoral condyle on the postoperative image

Based on previous research [9, 10] and the author's consideration, the reason for cross-pin protrusion may be related to the following points: (1) the direction of femoral tunnel insertion; (2) the direction of cross-pin insertion; and (3) the size of the femoral condyle. Therefore, measurements were obtained mainly to evaluate differences in these three parameters. All data were measured by the same person. Based on the above considerations, the following indicators were measured based on the postoperative MRI examination of each patient:

1. The angle between the femoral tunnel and Blumensaat's line (femoral tunnel angle): The median sagittal section of Blumensaat's line was selected, and the angle between the line of the midpoints of the two cross-pins and Blumensaat's line was measured (Fig. 2a).
2. The angle between the cross-pins and the line of the lowest point of the posterior side of the medial and lateral femoral condyles (cross-pin angle): The transverse section of the cross-pin was selected, and a straight line was drawn along the upper edge of the cross-pin and marked “cross-pin” (Fig. 2b). Then, the transverse section of lateral collateral ligament femoral insertion was selected, a line along the lowest point of the posterior medial and lateral femoral condyles was drawn, the line marked “cross-pin” for this section was copied, and the angle between them was measured (Fig. 2c).
3. The mediolateral diameter of the femoral condyle: The transverse section of lateral collateral ligament femoral insertion was selected, and a line parallel to the line along the lowest point of the posterior medial and lateral femoral condyles at the lateral collateral ligament femoral insertion point was drawn. The mediolateral diameter of the femoral condyle was then measured at this parallel line (Fig. 2d).
4. The anteroposterior diameter of the lateral femoral condyle: The transverse section of lateral collateral ligament femoral insertion was selected, and a vertical line was drawn to the line of the lowest point of the posterior medial and lateral femoral condyles at the largest anteroposterior diameter of the lateral femoral condyle. The anteroposterior diameter of the lateral femoral condyle was measured then at this vertical line (Fig. 2e).

### Surgical technique

**Fig. 3:**
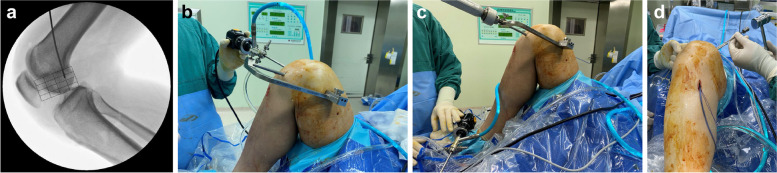
**a** According to the quadrant method [4], the accuracy of the femoral tunnel positioning was determined. **b** The Rigidfix femoral guide was placed reversely on the medial side of the knee. **c** The cross-pin tunnels were created from the medial side of the knee. **d** The cross-pins were inserted through the sleeves

Femoral tunnel preparation: With the knee at 120° flexion, the ACL femoral tunnel offset guide was inserted through the AM portal, and the ACL original femoral footprint was used as a landmark. The guide needle was positioned, and standard lateral fluoroscopy of the knee joint was performed during surgery. According to the quadrant method [11], we determined whether the femoral tunnel position was accurate (if the position was inaccurate, the needle entry point was adjusted, and fluoroscopy was performed again) (Fig. 3a). After confirming that the femoral tunnel position was correct, a calibrated full-thickness cannulated drill was used to make a femoral tunnel with a length of 30 mm along the direction of the positioning needle. The Rigidfix femoral guide was inserted into the femoral tunnel through the AM portal. The guide frame was rotated to the medial side of the knee at an angle of 20–40° relative to the horizontal (Fig. 3b), and the Rigidfix sleeves were drilled into the medial side of the femur, continuing until the hub met the guide (Fig. 3c). A guide pin was inserted into the sleeves, and the guide pin was confirmed by arthroscopy to accurately pass through the centre of the femoral tunnel.

Femoral graft fixation: With the knee at 90° flexion, after introducing the graft into the femoral tunnel, it was confirmed that the 30-mm mark of the graft was flush with the edge of the femoral tunnel. The Rigidfix cross-pins(42 mm) were inserted through the sleeves, maintaining tension in the proximal suture that exited through the skin of the thigh (Fig. 3d).

### Statistical analysis

Statistical analysis was performed using SPSS version 21.0, with the level of statistical significance set at *P* < 0.05. Descriptive data analysis (mean, standard deviation, range and proportion) was conducted for the entire patient population. The baseline characteristics of patients and demographic variables were compared between the groups using Student’s t test for variables and the chi-square test or exact Fisher test for proportions. A population distribution map was drawn for all cases according to the mediolateral diameter of the femoral condyle and the anteroposterior diameter of the lateral femoral condyle.

## Results

A total of 312 patients met the inclusion criteria, and 36 (11.5%) were lost to follow-up; There were 193 males and 83 females aged 14–58 years. There were 64 cases in the protrusion positive group and 212 cases in the protrusion negative groups. The proportion of cross-pin protrusion cases was 23.19% (64/276). Sixteen patients (5.79%) had chondral injury at the edge of the posterolateral femoral condyle. Five patients (1.81%) experienced postoperative dysfunction because of cross-pin protrusion. No patients experienced vascular or nerve injury.

Patient characteristics and demographic data are summarized in Table 1. There was no significant difference between the groups with respect to age, postoperative Lysholm score, Tegner score or knee laxity measurements. A significant difference was observed with respect to the sex distribution (*P* < 0.001).

**Table 1:** Patient characteristics and demographic data

	Protrusion positive group(*n* = 64)	Protrusion negative group(*n* = 212)	*P* value
Sex, n (%)			< 0.001
Male	1 (1.6)	192 (90.6)	
Female	63 (98.4)	20 (9.4)	
Age (years)	31.7 ± 11.5	29.3 ± 9.3	0.123
Follow-up (months)	14.5 ± 2.1	14.5 ± 1.8	0.949
Postoperative side-to-side laxity, mm	1.0 ± 0.8	1.0 ± 0.7	0.929
Tegner score	6.3 ± 1.0	6.5 ± 0.9	0.324
Lysholm score	95.6 ± 4.9	95.7 ± 4.6	0.825

Imaging data are summarized in Table 2. There was no significant difference between the groups with respect to the femoral tunnel angle or cross-pin angle. A significant difference was observed with respect to the mediolateral diameter of the femoral condyle and the anteroposterior diameter of the lateral femoral condyle (*P* < 0.001).

**Table 2:** Imaging data measurements

	Protrusion positivegroup(*n* = 64)	Protrusion negative group(*n* = 212)	*P* value
Femoral tunnel angle,	82.1 ± 6.0	82.8 ± 7.0	0.457
Cross-pin angle,	31.4 ± 9.1	30.6 ± 8.1	0.535
Mediolateral diameter of the femoral condyle, mm	70.6 ± 2.5 (65.9–73.16)	82.7 ± 4.2 (70.5–93.9)	< 0.001
Anteroposterior diameter of the lateral femoral condyle, mm	58.3 ± 2.9 (50.9–65.0)	66.4 ± 3.5 (59.52–78.18)	< 0.001

**Fig. 4:**
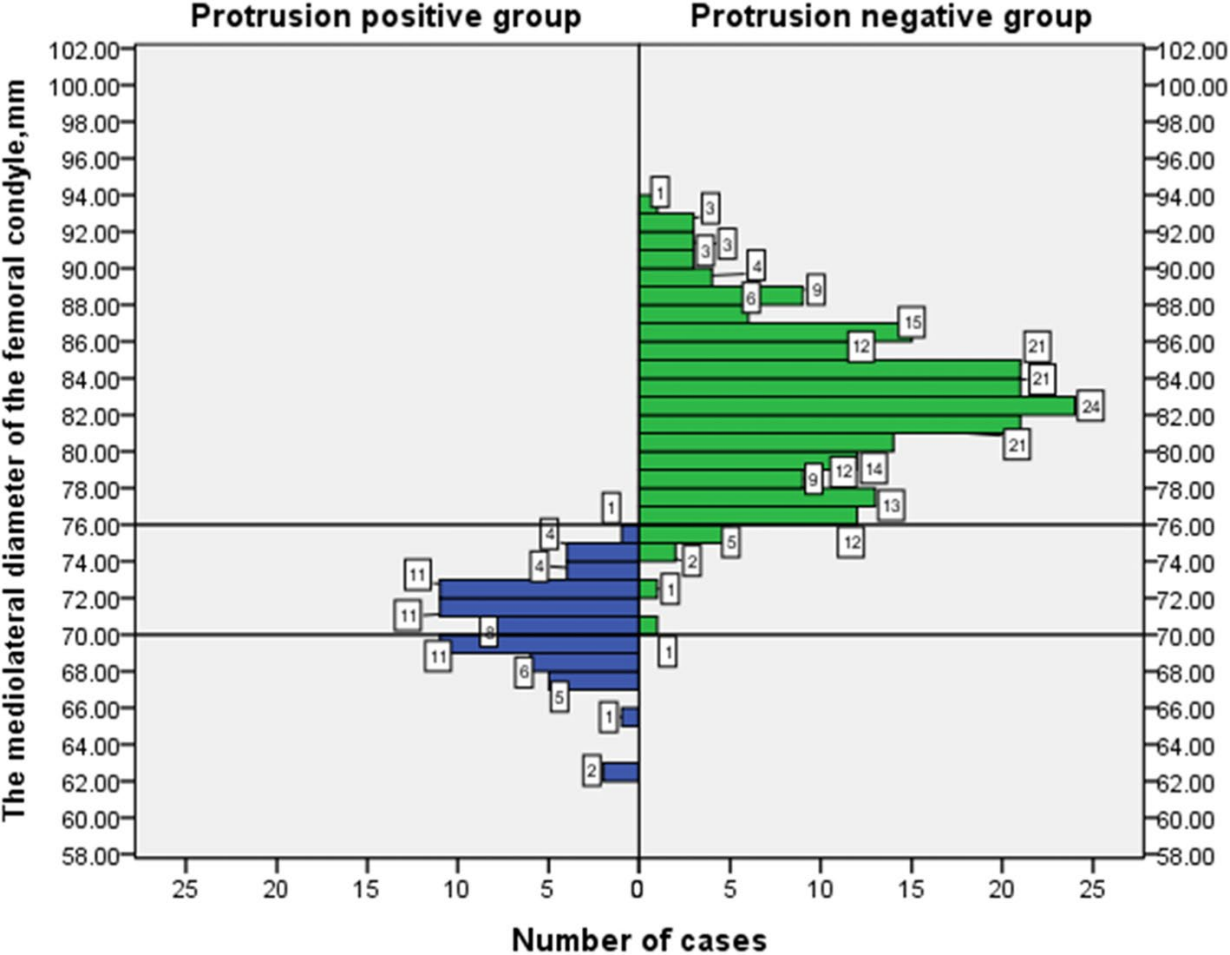
The population distribution map for all cases according to the mediolateral diameter of the femoral condyle

**Fig. 5:**
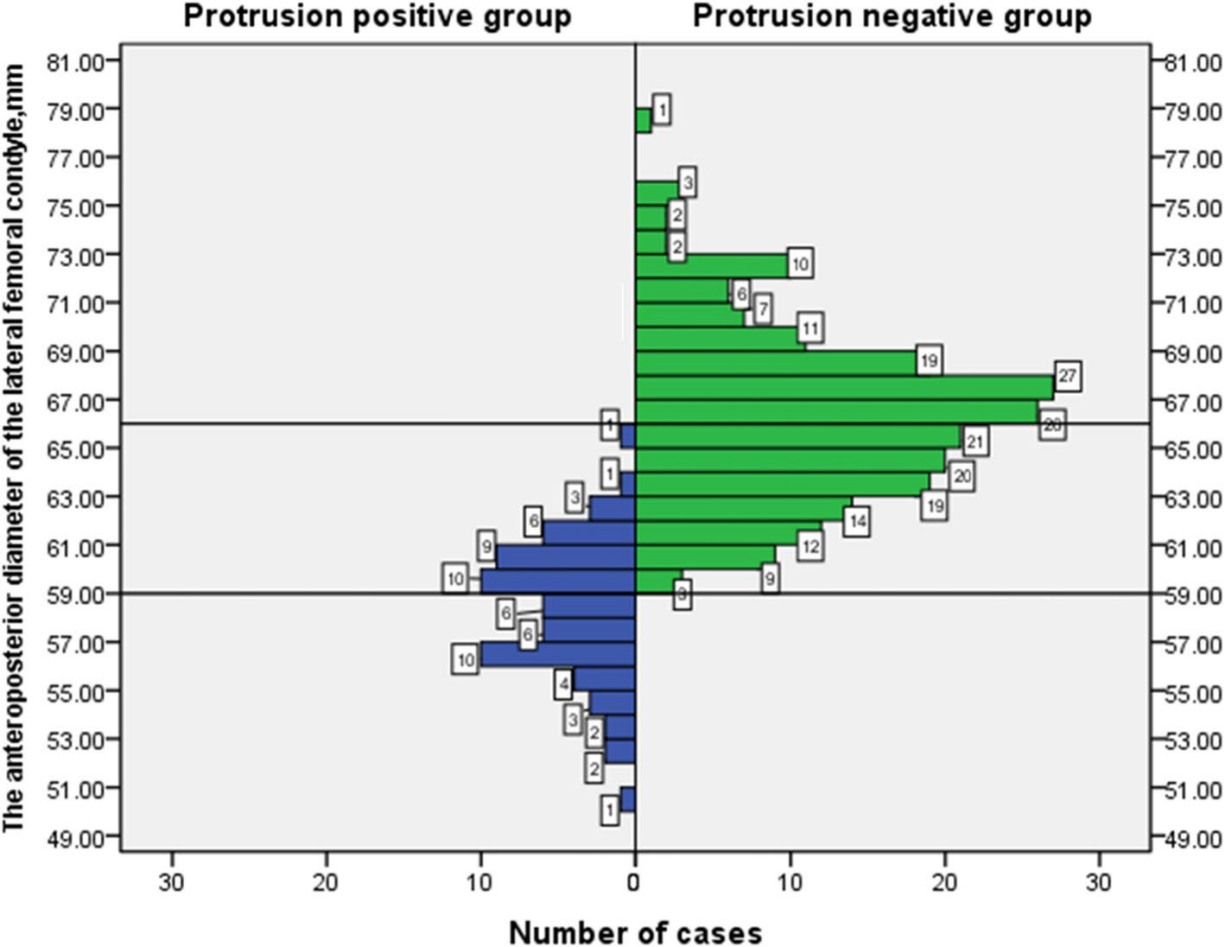
The population distribution map for all cases according to the anteroposterior diameter of the lateral femoral condyle

Figures 4 and 5 show the population distribution map for all cases according to the mediolateral diameter of the femoral condyle and the anteroposterior diameter of the lateral femoral condyle.

## Discussion

The main finding of this study is that the patients with cross-pin protrusion after treatment with the reverse Rigidfix femoral fixation device to prepare the femoral tunnel through the AM portal and fix the femur with the cross-pins (Rigidfix) in ACLR were mainly women with small femoral condyles. According to Figs. 4 and 5, the cross-pin penetration rate was 0% among patients with a mediolateral femoral condyle diameter ≥ 76 mm, and the cross-pin penetration rate was 100% among patients with a mediolateral femoral condyle diameter ≤ 70 mm. The cross-pin penetration rate was 0% among patients with an anteroposterior lateral femoral condyle diameter ≥ 66 mm, and the cross-pin penetration rate was 100% among patients with an anteroposterior lateral femoral condyle diameter ≤ 59 mm. The cross-pin penetration rate was 81.25% among patients with a mediolateral femoral condyle diameter of 70–76 mm, and the cross-pin penetration rate was 23.44% among patients with an anteroposterior lateral femoral condyle diameter of 59 mm-66 mm.

The fixed length of the cross-pin (Rigidfix) is 42 mm, and the author believes that the main reasons for these differences are as follows: (1) Although the mediolateral diameter of the femoral condyle and the anteroposterior diameter of the lateral femoral condyle were measured, each patient had obvious individual differences in the shape of the femoral condyle, so these measurements could not fully summarize the morphological characteristics of each patient's femoral condyle. (2) Cross-pin protrusion is closely related to the direction of femoral tunnel insertion and cross-pin insertion in each patient. (3) Measurement error: When the difference between the mediolateral diameter of the femoral condyle and the anteroposterior diameter of the lateral femoral condyle is small, the shape of the femoral condyle and the direction of femoral tunnel insertion and cross-pin insertion will influence the results. In the repeated measurement of the imaging data for patients in the case group, when the entrance to the femoral bone tunnel was determined for patients with relatively small femoral condyles (mediolateral femoral condyle diameter ≤ 70 mm, anteroposterior lateral femoral condyle diameter ≤ 59 mm), the distance between the centre point of the femoral tunnel (the midpoint of the cross-pins) and the cortex of the lateral femoral condyle was less than 21 mm (the length of the Rigidfix cross-pin is 42 mm) and the cross-pin inevitably penetrated the cortex when the entrance to the femoral bone tunnel was determined, regardless of how the angle changed.

In surgery for ACLR using a hamstring tendon graft, the best method of femoral graft fixation remains a matter of debate [4]. Enlargement of the femoral bone tunnel is particularly common in ACLR using hamstring grafts. Past biomechanical studies and clinical studies have found that the "bungee effect" and "wiper effect" are the main reasons for this outcome [12–15]. As the fixed point is closer to the tunnel entrance in the cross-pin method, this method can effectively reduce the "bungee effect" and "wiper effect". Compared with interference screw fixation and extracortical fixation, cross-pin (Rigidfix) fixation can effectively reduce the incidence of enlargement of the femoral bone tunnel [16–19]. Although cross-pins (Rigidfix) have obvious advantages in this respect, the Rigidfix femoral fixation device also has an obvious disadvantage in positioning the femoral tunnel. Positioning the femoral tunnel has always been the key to ACLR. Correct positioning of the tunnel is essential for restoration of the original physiological function of the ACL after ACLR [20]. The Rigidfix femoral fixation device is designed to use the TT technique to prepare the femoral tunnel, but due to the limitation of the tibial tunnel, it is difficult to obtain better femoral tunnel positioning. Shin et al. [21] proved that compared with the AM technique, femoral tunnels located by the TT technique tend to be high and anterior and have a certain distance from the commonly used femoral positioning points ("isometric" points or "anatomical" points). The reconstructed ACL is obviously different from the normal ACL, and it has obvious deficiencies in controlling tibial rotation. Moreover, other studies have shown that the knee joint after ACLR exhibits better rotational stability by femoral tunnel positioning by the AM technique than by the TT technique [22–26]. Therefore, some scholars have tried to use the cross-pin femoral fixation device to prepare the femoral tunnel through the AM portal [3, 23, 27–30], but iatrogenic injury caused by cross-pin tunnels prepared from the outside to the inside is major, especially chondral injury. Castoldi et al. [9] found through a cadaver study using the Rigidfix system to prepare a femoral tunnel through the AM portal that as the insertion angle of the pin increases, the risk of chondral injury increases from 80 to 100%. Inácio et al.[28] also found that in postoperative CT three-dimensional reconstruction after Rigidfix femoral fixation through the AM portal for ACLR, the chondral injury rate was as high as 49.99%. However, what is more dangerous is that with the increase in the insertion angle of the femoral tunnel, the cross-pins may penetrate from the posterior cortex to the popliteal fossa, causing damage to important vessels and nerves in the popliteal fossa [10]. Therefore, most scholars do not recommend using the Rigidfix femoral fixation device to prepare femoral tunnels through the AM portal. The reverse placement of the Rigidfix femoral fixation device to prepare the femoral tunnel through the AM portal effectively reduces iatrogenic injury while providing better femoral tunnel positioning than the traditional TT technique [8]. Our department has found through clinical practice that although this technique is effective in ACLR, a small number of patients show cross-pin penetration of the lateral femoral condyle cortex, with some even penetrating the cartilage, resulting in chondral injury. In severe cases, this penetration can cause injury to the popliteal tendon or posterolateral joint capsule, which can lead to discomfort during movement of the knee joint and reduced range of motion in the early postoperative period. Therefore, this study was conducted. To the best of our knowledge, this is the first clinical study that has identified and analysed cross-pin protrusion after the use of this technique for ACLR. This technique poses a safety hazard in patients with relatively small femoral condyles. Based on the analysis of a large amount of clinical imaging data, the safety range of the femoral condyle for the use of this technology is proposed; using this range could effectively improve the clinical safety and practicality of this technology and provide clinicians with a safer and more effective ACLR technology.

Although the cross-pin protrusion rate of this technique was 21.19%, the position of cross-pin penetration was mostly behind the popliteal tendon and the lateral collateral ligament femoral insertion point. Only 16 patients (5.79%) had chondral damage at the edge of the posterolateral femoral condyle. Moreover, a large proportion of patients had a cross-pin penetration length of approximately 1–2 mm, and 271 patients (98.19%) had no symptoms after surgery; additionally, there was no impact on postoperative recovery. Only 5 patients (1.81%) experienced discomfort during flexion and extension around the femoral insertion point of the popliteal tendon after surgery, but there was no obvious pain, and the range of knee joint motion was reduced in the early postoperative period. The range of knee joint flexion just reached 70–90° within 8 weeks after surgery. Through postoperative MRI and CT examination, approximately 4–5 mm of the cross-pins was exposed from the femoral condylar cortex, which directly injured the popliteal tendon or posterolateral joint capsule (Fig. 1d). During the follow-up period, the symptoms of patients with discomfort caused by cross-pin protrusion completely disappeared within 3 months after the operation, with no impact on the stability of the ACL or the recovery of knee function after reconstruction. However, further scientific research is needed. None of the 276 patients experienced any adverse events, such as vascular or nerve injury, rupture or cross-pin failure. Compared with traditional Rigidfix femoral fixation to prepare the femoral tunnel, whether with the TT technique or the AM technique, the present approach significantly reduces the frequency of iatrogenic injury and increases safety. Before surgery, the mediolateral diameter of the femoral condyle and the anteroposterior diameter of the lateral femoral condyle can be measured with MRI to assess the risk of cross-pin protrusion and the suitability of this ACLR technique for the patient. After preoperative evaluation, there may be a risk of cross-pin protrusion, but this technology can still be applied if desired. According to clinical experience, the hub is kept 5 mm from the guide when drilling the Rigidfix sleeves, and the same distance is reserved for asymmetric cross-pin fixation when the cross-pins are placed. For patients with relatively small femoral condyles (mediolateral femoral condyle diameter ≤ 70 mm, anteroposterior lateral femoral condyle diameter ≤ 59 mm), this technique is not recommended.

## Conclusions

The patients with cross-pin protrusion after treatment with the reverse Rigidfix femoral fixation device to prepare the femoral tunnel through the AM portal and fix the femur with cross-pins (Rigidfix) in ACLR were mainly women with small femoral condyles. For patients with a mediolateral femoral condyle diameter ≥ 76 mm and an anteroposterior lateral femoral condyle diameter ≥ 66 mm, there is no risk of cross-pin protrusion, so this technique can be used with confidence.

## Supplementary Information

**Additional file 1. **

**Additional file 2. **

## Data Availability

The datasets generated during and analyzed during the current study are not publicly available due to privacy and ethical restrictions but are available from the corresponding author on reasonable request.

## Availability of data and materials

The datasets generated during and analyzed during the current study are not publicly available due to privacy and ethical restrictions but are available from the corresponding author on reasonable request.
